# Supplementary material for: Comparison performance of the Bayesian Approach with the Weibull and Birnbaum-Saunders distributions in imputation of time-to-event censors
Source: PLoS One. 2024 Jan 22;19(1):e0295977. doi: 10.1371/journal.pone.0295977 (PMC10802968; doi:10.1371/journal.pone.0295977)
Supplement: S1 Table — (DOCX) [file pone.0295977.s001.docx]

**Supporting Files**

**S1 Table**. Values of the parameter 𝜃 of exponential distribution for different percentages of censoring and different values of parameters of Weibull distribution

| Censoring Percent  Weibull Parameter | 0.10 | 0.20 | 0.50 |
| --- | --- | --- | --- |
| W(0.5. 4) | 0.02 | 0.04 | 0.30 |
| W(1.4) | 0.04 | 0.08 | 0.30 |
| W(2,4) | 0.02 | 0.06 | 0.20 |
