# Supplementary material for: Comparison performance of the Bayesian Approach with the Weibull and Birnbaum-Saunders distributions in imputation of time-to-event censors
Source: PLoS One. 2024 Jan 22;19(1):e0295977. doi: 10.1371/journal.pone.0295977 (PMC10802968; doi:10.1371/journal.pone.0295977)
Supplement: S2 Table — (DOCX) [file pone.0295977.s002.docx]

**Supporting Files**

**S2 Table**. Values of regression coefficients for different values of Weibull distribution shape parameter

| Coefficient  Shape | $b_{1}$ | $b_{2}$ |
| --- | --- | --- |
| 0.5 | -0.8 | 0.2 |
| 1 | -1.5 | 0.40 |
| 2 | -3 | 0.3 |
