# Supplementary material for: Comparison performance of the Bayesian Approach with the Weibull and Birnbaum-Saunders distributions in imputation of time-to-event censors
Source: PLoS One. 2024 Jan 22;19(1):e0295977. doi: 10.1371/journal.pone.0295977 (PMC10802968; doi:10.1371/journal.pone.0295977)
Supplement: S4 Table — (DOCX) [file pone.0295977.s004.docx]

**Supporting Files**

**S4 Table**. Values of regression coefficients for different values of Birnbaum-Saunders distribution shape parameter.

| Censoring Percent  BS Parameter | 10% | | | 20% | | | 50% | | |
| --- | --- | --- | --- | --- | --- | --- | --- | --- | --- |
|  | N=100 | N=200 | N=300 | N=100 | N=200 | N=300 | N=100 | N=200 | N=300 |
| BS (0.5. 4) | 0.01 | 0.02 | 0.03 | 0.04 | 0.04 | 0.05 | 0.15 | 0.15 | 0.15 |
| BS (1.4) | 0.02 | 0.01 | 0.02 | 0.04 | 0.04 | 0.04 | 0.15 | 0.15 | 0.15 |
| BS (2,4) | 0.01 | 0.01 | 0.01 | 0.02 | 0.02 | 0.02 | 0.10 | 0.15 | 0.15 |
