# Supplementary figures and images for: Comparison performance of the Bayesian Approach with the Weibull and Birnbaum-Saunders distributions in imputation of time-to-event censors
Source: PLoS One. 2024 Jan 22;19(1):e0295977. doi: 10.1371/journal.pone.0295977 (PMC10802968; doi:10.1371/journal.pone.0295977)

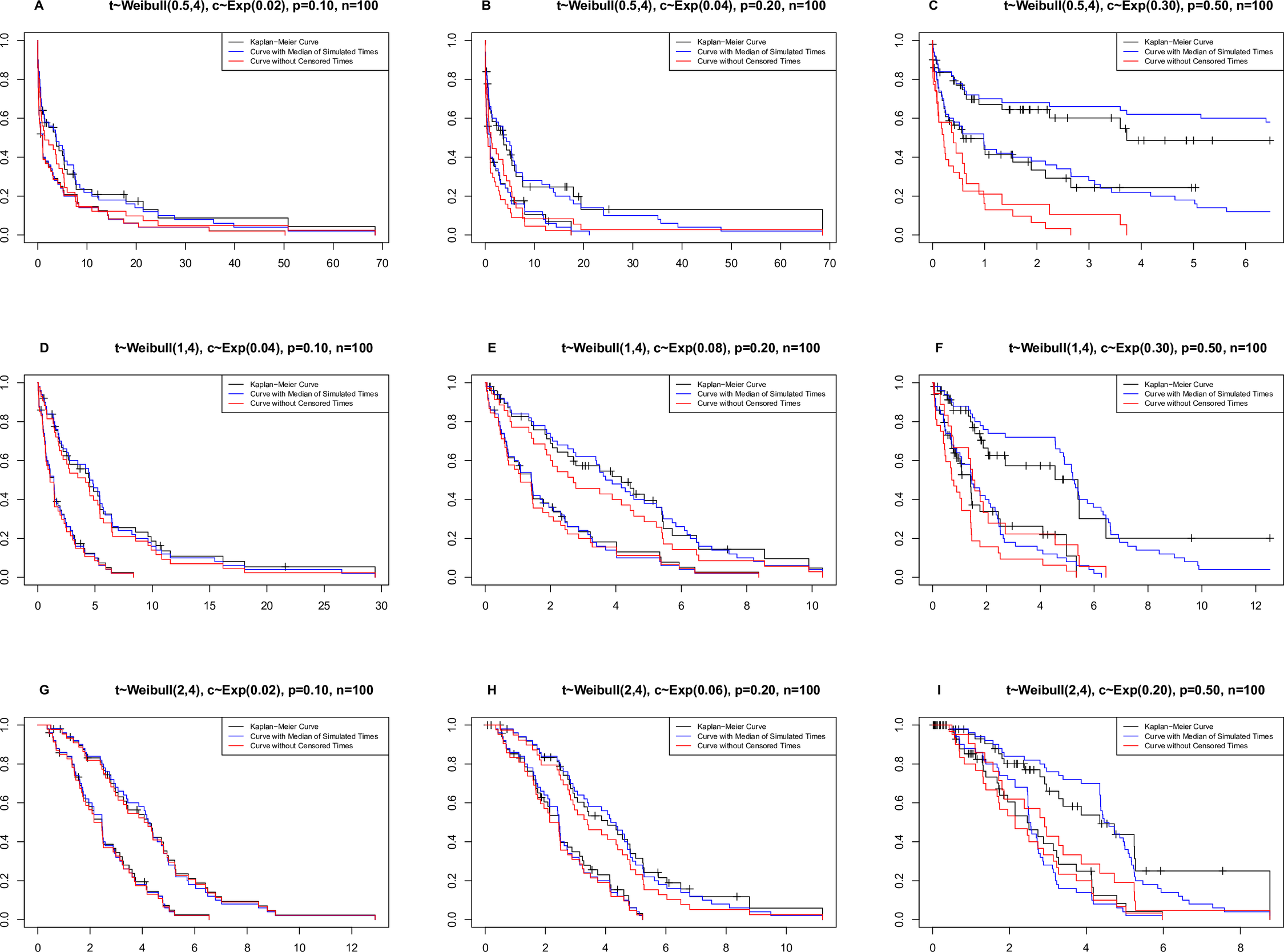

Supplement: S1 Fig — A) t~weibull(0.5,4), c~Exp(0.02), 10% censoring, and 100 sample sizes. B) t~weibull(0.5,4), c~exp(0.04), 20% censoring, and 100 sample sizes. C) t~weibull(0.5,4), c~Exp(0.30), 50% censoring, and 100 sample sizes. D) t~weibull(1,4), c~Exp(0.04), 10% censoring, and 100 sample sizes. E) t~weibull(1,4), c~exp(0.08), 20% censoring, and 100 sample sizes. F) t~weibull(1,4), c~Exp(0.30), 50% censoring, and 100 sample sizes. G) t~weibull(2,4), c~Exp(0.02), 10% censoring, and 100 sample sizes. H) t~weibull(2,4), c~exp(0.06), 20% censoring, and 100 sample sizes. I) t~weibull(2,4), c~Exp(0.20), 50% censoring, and 100 sample sizes. (TIF) [file pone.0295977.s005.tif]

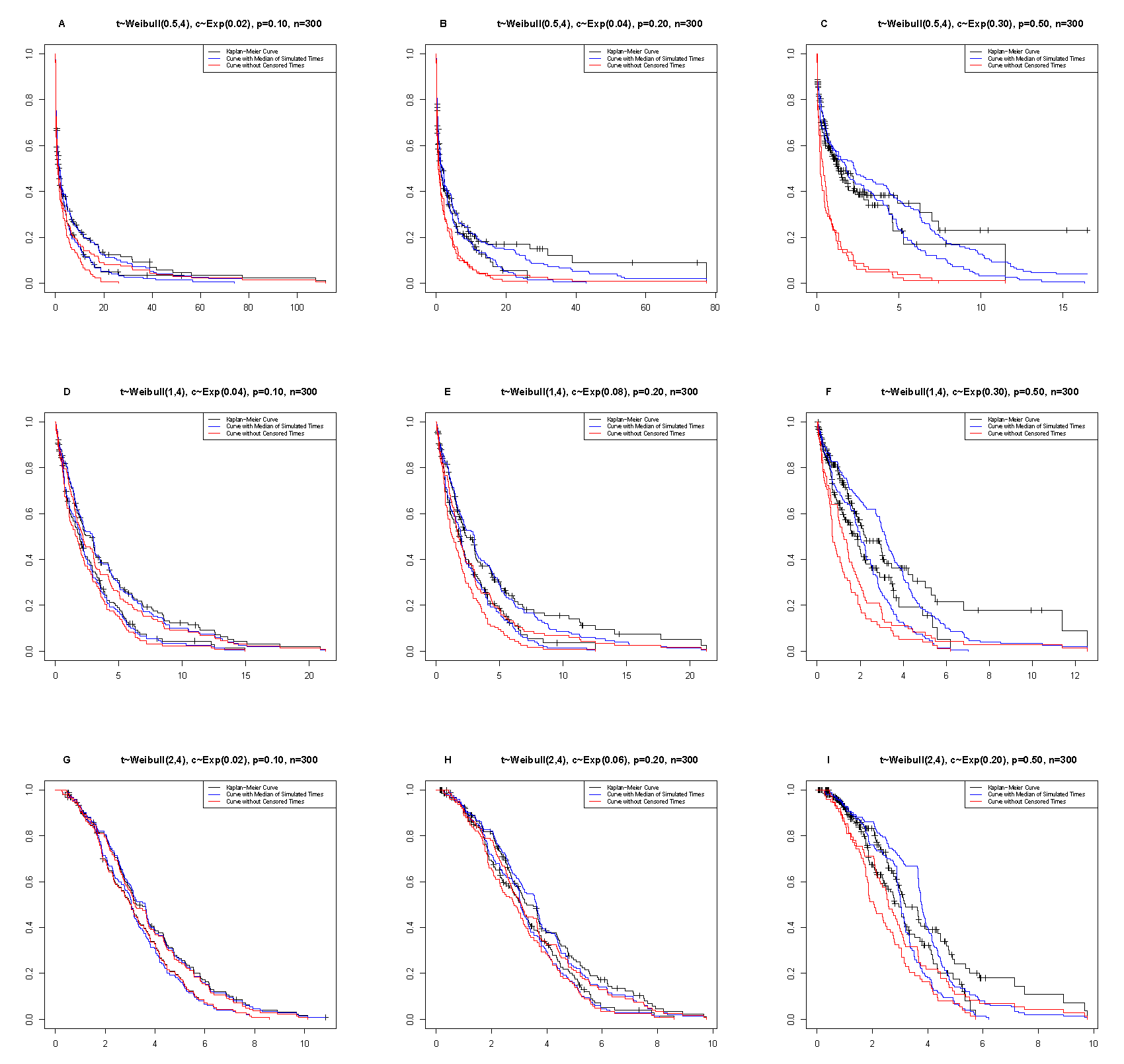

Supplement: S2 Fig — A) t~weibull(0.5,4), c~Exp(0.02), 10% censoring, and 300 sample sizes. B) t~weibull(0.5,4), c~exp(0.04), 20% censoring, and 300 sample sizes. C) t~weibull(0.5,4), c~Exp(0.30), 50% censoring, and 300 sample sizes. D) t~weibull(1,4), c~Exp(0.04), 10% censoring, and 300 sample sizes. E) t~weibull(1,4), c~exp(0.08), 20% censoring, and 300 sample sizes. F) t~weibull(1,4), c~Exp(0.30), 50% censoring, and 300 sample sizes. G) t~weibull(2,4), c~Exp(0.02), 10% censoring, and 300 sample sizes. H) t~weibull(2,4), c~exp(0.06), 20% censoring, and 300 sample sizes. I) t~weibull(2,4), c~Exp(0.20), 50% censoring, and 300 sample sizes. (TIF) [file pone.0295977.s006.tif]

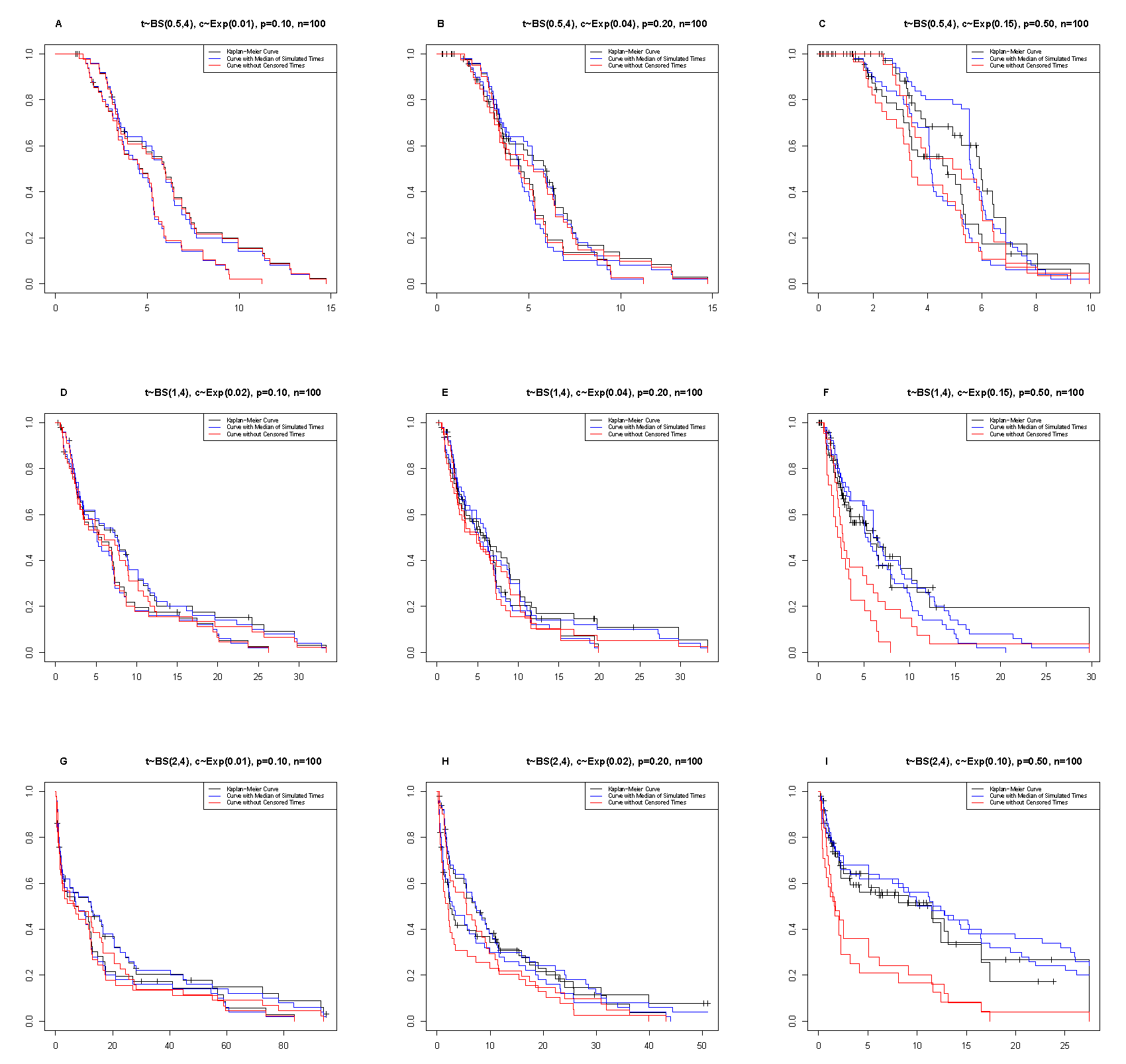

Supplement: S3 Fig — A) t~BS(0.5,4), c~Exp(0.01), 10% censoring, and 100 sample sizes. B) t~BS(0.5,4), c~exp(0.04), 20% censoring, and 100 sample sizes. C) t~BS(0.5,4), c~Exp(0.15), 50% censoring, and 100 sample sizes. D) t~BS(1,4), c~Exp(0.02), 10% censoring, and 100 sample sizes. E) t~BS(1,4), c~exp(0.04), 20% censoring, and 100 sample sizes. F) t~BS(1,4), c~Exp(0.15), 50% censoring, and 100 sample sizes. G) t~BS(2,4), c~Exp(0.01), 10% censoring, and 100 sample sizes. H) t~BS(2,4), c~exp(0.02), 20% censoring, and 100 sample sizes. I) t~BS(2,4), c~Exp(0.10), 50% censoring, and 100 sample sizes. (TIF) [file pone.0295977.s007.tif]

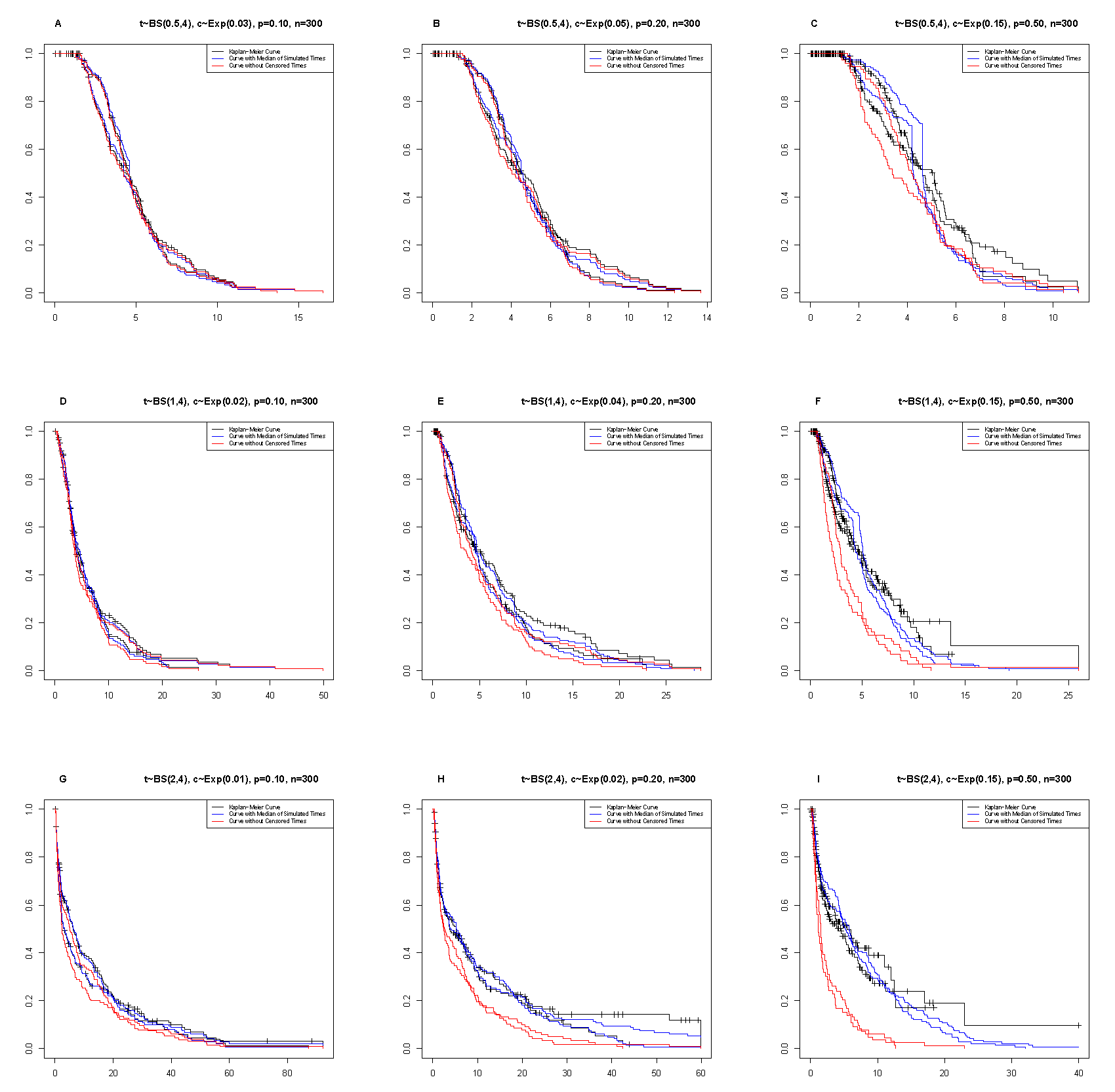

Supplement: S4 Fig — A) t~BS(0.5,4), c~Exp(0.03), 10% censoring, and 300 sample sizes. B) t~BS(0.5,4), c~Exp(0.05), 20% censoring, and 300 sample sizes. C) t~BS(0.5,4), c~Exp(0.15), 50% censoring, and 300 sample sizes. D) t~BS(1,4), c~Exp(0.02), 10% censoring, and 300 sample sizes. E) t~BS(1,4), c~Exp(0.04), 20% censoring, and 300 sample sizes. F) t~BS(1,4), c~Exp(0.15), 50% censoring, and 300 sample sizes. G) t~BS(2,4), c~Exp(0.01), 10% censoring, and 300 sample sizes. H) t~BS(2,4), c~Exp(0.02), 20% censoring, and 300 sample sizes. I) t~BS(2,4), c~Exp(0.15), 50% censoring, and 300 sample sizes. (TIF) [file pone.0295977.s008.tif]
