# Supplementary material for: Comparison performance of the Bayesian Approach with the Weibull and Birnbaum-Saunders distributions in imputation of time-to-event censors
Source: PLoS One. 2024 Jan 22;19(1):e0295977. doi: 10.1371/journal.pone.0295977 (PMC10802968; doi:10.1371/journal.pone.0295977)
Supplement: S1 File — (DOCX) [file pone.0295977.s009.docx]

**Supporting Files**

**WibBUGS and R codes:**

OpenBUGS (Bayesian inference Using Gibbs Sampling) program was used to impute censored observations. For simulation and impute, it is necessary to enter the data into the OpenBUGS in a different way. Observed times (i.e, y_i) is transformed into two variables: time to censoring (c) and time to event (t) with the censoring indicator as NA and is categorized as follows.

$t_{i}= \left\{ \begin{aligned} \begin{aligned} y_{i}, if \delta_{i}=1 \\ NA, if \delta_{i}=0 \end{aligned} c_{i}= \left\{ \begin{aligned} y_{i}, if \delta_{i}=0 \\ 0, if \delta_{i}=1 \end{aligned} \right. \\ \end{aligned} \right.$

The samples in OpenBUGS are generated from the posterior distribution based on the probabilistic idea of a Markov Chain with a distribution corresponding to the desired posterior distribution. Censorship is observed in most of the data and defined in the distribution of times. Assume time to event distribution with the parameter θ, then for interval, right and left censoring respectively, distribution is defined as follows:

$$t_{i}\sim dist\left( \theta\right)I\left( a,b \right), a<t<b; t_{i}\sim dist\left( \theta\right)I\left( a, \right); t_{i}\sim dist\left( \theta\right)I\left( ,b \right)$$

Accordingly, when OpenBUGS receives the time to event as NA, function related to right censoring added to the likelihood function (f(t; θ)). Assuming that right censored observed times have Weibull distribution , the model is written in the OpenBUGS as follows (1,2):

GitHub Link to Codes:

<https://github.com/pshkhoei/Thesis-Paper-1>

Four scenarios' command codes include:

1. Simulation of censored times with the Weibull distribution.

2. Simulation of censored times with the Birnbaum-Saunders distribution.

3. Simulation of censored times in the Breast Cancer dataset with the Weibull distribution.

4. Simulation of censored times in the Breast Cancer dataset with the Birnbaum-Saunders distribution.

The R-markdown result (as HTML and Pdf) obtained by running the command codes was uploaded to GitHub page. The output data from scenarios 1 and 2 was uploaded to GitHub, and the data on breast cancer from scenarios 3 and 4 was placed in a text file with the name Data-Paper1 and submitted to PLOS ONE as other file.

References:

1. Lunn D, Jackson C, Best N, Thomas A, Spiegelhalter D. The BUGS Book: A Practical Introduction to Bayesian Analysis. Boca Raton, FL, USA: CRC Press; 2012.

2. Sturtz S, Ligges U, Gelman A. R2OpenBUGS: A Package for Running OpenBUGS from R. 3.2-3.2.1 ed2020.
