# Supplementary material for: Comparison performance of the Bayesian Approach with the Weibull and Birnbaum-Saunders distributions in imputation of time-to-event censors
Source: PLoS One. 2024 Jan 22;19(1):e0295977. doi: 10.1371/journal.pone.0295977 (PMC10802968; doi:10.1371/journal.pone.0295977)
Supplement: Attachment — Submitted filename: Responce to reviewers.docx [file pone.0295977.s010.docx]

02/09/2023

Manuscript PONE-D-23-19782

Response to Reviewers

Dear Dr. Khorasani, Academic Editor

Thank you for giving us the opportunity to submit a revised draft of the manuscript “Comparison of the Performance of Bayesian Approach with Weibull and Birnbaum-Saunders Distributions in Imputation of Time-to-Event Censors” for publication in the Journal of PLOS ONE. We appreciate the time and effort that you and the reviewers dedicated to providing feedback on our manuscript and are grateful for the insightful comments on and valuable improvements to our paper.

We have incorporated most of the suggestions made by the reviewers. Those changes are highlighted within the manuscript. Please see below, in blue, for a point-by-point response to the reviewers’ comments and concerns. All line numbers refer to the revised manuscript file with tracked changes.

Reviewers' Comments to the Authors:

Reviewer 1

1. The novelty of the article is unclear as why Weibull and Birnbaum-Saunders distributions are focused?

Author's response: Thank you, dear reviewer:

This analysis is innovative because it compares the performance of two distributions in the presence of missing data. The importance of Birnbaum-Saunders distribution is discussed in Sections 2-3. It is stated that:

- Fatigue Life data is modeled using Birnbaum-Sanders.

- Lifespan and sickness in humans are similar to the lifespan and failure of parts for vehicles. This distribution can therefore be used as a model for the length of patient's illness. Every part of a person wears out over time, just like a part of a vehicle. We may therefore discuss the loss or degradation of human organ function in the same way that we can discuss the use of this distribution to predict the wear and tear of vehicle components.

- Because the normal distribution is one of the most useful distributions under research, the Birnbaum-Saunders distribution provides exceptional flexibility because it converges to the normal distribution under certain parameters.

- The most significant and popular distribution in survival analysis is the Weibull distribution.

This has led the authors of this study to compare the Birnbaum-Sanders distribution's validity to the Weibull distribution.

2. There are many typos and grammatical mistakes which must be fixed carefully.

Author's answer: You have mentioned the right point. In light of this, the study's entire text has been altered and paraphrased. Grammatical mistakes were fixed. To keep track of the changes, follow the attached file, please.

3. On page 5, each equation must be written separately.

Author's response: Absolutely accurate. Each formula was put on one line (lines 253–256).

4. The presentations of figures must be improved and interpret each figure.

Author's response: I appreciate you bringing up that point. All of the images have been redrawn, and labels have been added that explain each curve in more detail. Following are the lines that describe each figure:

Figure 1: Lines 264 to 266.

Figure 2: Lines 299 to 300.

Figure 3: In order to show the associated explanations for the shapes independently, the shape parameters, censoring percent, and sample size were  kept fix. There are explanations in lines 556 to 564.

Figure 4: Lines 569 to 575.

Figure 5: Lines 593 to 601.

Figure 6: Lines 613 to 615.

Figure 7 and 8: Lines 665 to 673.

5. How the present work is different in terms of Bayesian analysis than the many studies available in the literature.

Author's response: Many thanks. Following are the distinctions:

1. A comprehensive explanation of the working procedure is provided along with the formula.

2. Bayesian analysis has been applied to two distinct distributions.

3- The median and average of the simulated numbers were used to assess how well the distributions worked.

4- Command codes for Bayesian analysis are included with the article.
6. The real data set must be properly described.

Author's response: Thank you.

The real data description section was expanded and grammar and sentence errors were corrected (lines 626 to 648).

7. It is not clear how the Bayesian analysis is conducted. There are no graphical diagnostic tests reported in the text.

Author's response: Thank you.

In lines 473 to 506, the Bayesian analysis procedure is explained. For a sample size of 200 numbers, the following procedures are implemented in the R command codes:

1- Produce 200 data with a probability of 50% for the covariate x.

2- Setting the distributions' shape and scale parameters to the appropriate values (Tables 1 to 4).

3- Produce 200 data using the Birnbaum-Sanders or Weibull distribution.

4- Generating 200 data from the exponential distribution with the parameters reported in Tables 1 and 3, so that its censoring percentage is equal to the values of 10, 20 and 50%.

5- Merging the data from sections 3 and 4, to create a unit data set:

The data with a lower size is chosen for the merge. The output of this section will be 200 data, some of which will be censored according to the wanted percentage, and the remaining data will be drawn from the Weibull (Birnbaum-Sanders) distribution.

6- Simulation of the censored values in the data of section 5 using the R2OpenBUGs ® package in the number of 11,000 times, of which about 1,000 data have been burned and eventually 10,000 simulated data will remain for each censored.

7- Replace any censored data in the section 5 with the mean or median of the 10,000 simulated data from section 6, instead of to that data.

8- Using the information from section 5 to create the Kaplan-Meier curve.

9- Removing the data from section 5 that was censored and plotting their curve.

10 Draw a curve for each percent of censoring using 10,000 simulated for every censoring and related observed data points.

11- Draw a curve that includes the median of 10,000 simulated data points and the observed data.

12- Create a curve using the average of 10,000 simulated data points as well as the observed data.

13- Comparing two distributions' performance using the DIC index obtained from Bayesian analysis.

Reviewer 2

1. Page 3, Introduction Section: I don’t think it is correct to say that “it is necessary to impute the incomplete data”. Many survival models do not use the imputation approach. For example, the Cox proportional hazards model is established under the framework of counting processes and martingales.
Author's response: Many thanks.

The explanation of this statement has been expanded with lines 72 to 77. To support the reasoning, more than three sources were added.

2. I think Sections 2, 3.1, and 3.2 are too long. The description of the literature review can be more concise. Sections 3.1 and 3.2 are very basic so they can be described more concisely as well.
Author's response: I appreciate the point you made:

- The section that concentrated on the literature review was studied. Reference number 17, which has to do with imputed values in time series data, was removed from lines 171 to 175 due to the nature of the study's data.

- Since the explanations in sections 3.1 and 3.2 are related to different functions and calculation formulas used in Bayesian analysis, omitting or condensing them would undermine the study's overall purpose. The rationale for selecting the Birnbaum-Sanders distribution is also presented in section 3.2. As a result, nothing changed.

3. In the simulation studies, does the generation of censoring time include administrative censoring? If not, it will be helpful to include such a situation.
Author's response: I appreciate your point.

The right data censoring type was used in this investigation. However, theoretically, there is no restriction on the kind of censoring, and with a small adjustment, the method can be applied to data that has been left-censored and interval-censored.

4. In evaluating the performance of the proposed method, is it of interest to investigate the results of statistical inference (such as bias, standard error, empirical standard deviation, coverage of 95% confidence interval, etc.) based on the imputed data? If not, why not? If yes, it will be helpful to add these results.

Author's response: Many thanks.

It is necessary to explain the point.

There is no comprehensive standard for validation and verification in the simulation of censored data, according to an assessment of the literature (1). Methods for verification and validation have been explored in Edmonds et al. (2). Validation for this study was carried out in a similar way, including:

1. Making use of three different sample sizes (100, 200, and 300).

2- Using various distribution shape parameters (sensitivity analysis)

3. Using various censoring percentages (Sensitivity analysis).

4- Checking the results of the procedure using real data.

The following method has been tested for verification in this study:

1- Review and confirm of 56 scenarios using R and OpenBUGs command codes:

- Up to 27 Weibull distribution scenarios, with three variations for the shape parameter, three variations for the censoring percent, and three variations for the sample size.

- 27 scenarios for the Birnbaum-Saunders distribution, each with three possible values for the shape parameter, the censoring percent, and the sample size.

- Two possibilities for the Weibel and Birnbaum-Sanders distribution in real data

2- The execution of command codes on real data, including the Weibel and Birnbaum-Sanders distribution scenarios.

Lines 684 to 691 have been included to the research in this regard.

5. Please add some discussions about any potential future research directions.
Author's Response: I appreciate your insight.

This is discussed on lines 786 to 789. The simulation technique will be applied by the study's authors to the Bayesian network-based analysis of breast cancer data. An article has been written about it.

Sincerely.

**References**

1.	Sargent RG, Balci O, editors. History of verification and validation of simulation models. 2017 Winter Simulation Conference (WSC); 2017 3-6 Dec. 2017.

2.	David N, Fachada N, Rosa A.C. Simulating Social Complexity. In: Edmonds B, Meyer R, editors. 2nd ed. Berlin: Springer; 2017. p. 173-205.
