# Supplementary material for: Comparison performance of the Bayesian Approach with the Weibull and Birnbaum-Saunders distributions in imputation of time-to-event censors
Source: PLoS One. 2024 Jan 22;19(1):e0295977. doi: 10.1371/journal.pone.0295977 (PMC10802968; doi:10.1371/journal.pone.0295977)
Supplement: Attachment — Submitted filename: Responce to reviewers, 2023-11-30.docx [file pone.0295977.s011.docx]

11/30/2023

Manuscript PONE-D-23-19782

Response to Reviewers

Dear Editors

Again, thanks for giving us the opportunity to submit a revised draft of the manuscript “Comparison of the Performance of Bayesian Approach with Weibull and Birnbaum-Saunders Distributions in Imputation of Time-to-Event Censors” for publication in the Journal of PLOS ONE. We appreciate the time and effort that you and the reviewers dedicated to providing feedback on our manuscript and are grateful for the insightful comments on and valuable improvements to our paper.

We have incorporated all of the suggestions made by the reviewers. Those are in a “track change” within the manuscript. Please see below, in blue, for a point-by-point response to the reviewers’ comments and concerns.

Reviewers' Comments to the Authors:

Journal Requirements:

1. Please ensure that your manuscript meets PLOS ONE's style requirements, including those for file naming.

Author's response:

Thank you.

The paper has been modified in accordance with the style of Plus ONE.

2. Please note that PLOS ONE has specific guidelines on code sharing for submissions in which author-generated code underpins the findings in the manuscript.

Author's response:

Many thanks.

We have the following four scenarios and the codes were written for them.

1. Simulation of censored times with the Weibull distribution.

2. Simulation of censored times with the Birnbaum-Saunders distribution.

3. Simulation of censored times in the Breast Cancer dataset with the Weibull distribution.

4. Simulation of censored times in the Breast Cancer dataset with the Birnbaum-Saunders distribution.

The R codes and R-markdown result (as HTML and Pdf) obtained by running the command codes was uploaded to GitHub page. The output data from scenarios 1 and 2 was uploaded to GitHub, and the data on breast cancer from scenarios 3 and 4 was placed in a text file with the name Data-Paper1 and submitted to PLOS ONE as other file.

Note: Breast cancer data has been sent to the referees for verification.

GitHub Link:

<https://github.com/pshkhoei/Thesis-Paper-1>

3. We suggest you thoroughly copyedit your manuscript for language usage, spelling, and grammar.

Author's response:

Many thanks.

The article has been thoroughly reviewed and modified. Grammar and spelling mistakes were fixed.

4. In your Data Availability statement, you have not specified where the minimal data set underlying the results described in your manuscript can be found.

Author’s response:

Thanks.

The code and Breast cancer data have been uploaded.

GitHub Link for R codes:

<https://github.com/pshkhoei/Thesis-Paper-1>

5. Your ethics statement should only appear in the Methods section of your manuscript. If your ethics statement is written in any section besides the Methods, please move it to the Methods section and delete it from any other section.

Author’s response:

Thanks a lot. Done.

6. We note you have included a table to which you do not refer in the text of your manuscript. Please ensure that you refer to Table 4 in your text.

Author’s response:

Thank you very much. Explanations for the tables and reference to it were included in the text.
Reviewers’ comments:

Reviewer #1:

1. Have the authors made all data underlying the findings in their manuscript fully available?

Author’s response:

Thanks.

The code and Breast cancer data have been uploaded.

GitHub Link for R codes:

<https://github.com/pshkhoei/Thesis-Paper-1>

2. Is the manuscript presented in an intelligible fashion and written in standard English?

Author’s response:

Many thanks.

The article has been thoroughly reviewed and modified. Grammar and spelling mistakes were fixed.

3. On page 4, line 113, what is meant by 4ecommend Pratama et al. ?

Author’s response:

Thank you very much. Corrected.

4. References are not correctly cited. Please cite them carefully.

Author’s response:

Thanks. All of the references were double-checked, and any necessary changes were made.

5. Equations are not written properly. For example, the median survival equation does not look correct.

Author’s response:

Many thanks.

Every formula, particularly the median survival time, has been reviewed and updated.

6. To determine scale parameter, how the values of x are generated?

Author’s response:

Thanks a lot.

There are two possible states for the variable $x$: zero and one, with equal probability of 0.5. This variable is used in the scale parameter of the distributions of the survival time (Weibull and Birnbaum-Saunders). That is, instead of using a fixed values (say 4) for the scale parameter, the scale was linked to a two-state variable which represented two groups.

7. Weibull must be written with capital W.

Author’s response:

Thank you very much. Corrected.

8. I still cannot figure out why imputation of censored data because censoring mechanism is different from missing value?

Author’s response:

Thank you.

The Bayesian approach can be used to impute missing and censored data. The aim of this research is to apply the Bayesian method to impute censoring times. For this purpose, the computations in Sections 4.1 to 4.3 are written for censored times conditionally. Review, for instance, equations 5 through 7.

$$f\left( t | T\geq c, \theta\right)= \frac{f\left( t | \theta\right)}{S\left( c | \theta\right)}$$

$$E\left( T | T\geq c \right)= \frac{\int_{c}^{\infty} t f\left( t | \theta\right)dt}{S\left( c | \theta\right)}$$

$$0.5= S(T|T\geq c, \theta) =\frac{P(T\geq t_{med}|\theta)}{P(T\geq c|\theta)}=\frac{S(t_{med}|\theta)}{S(c|\theta)}$$

If imputation is used to fill in missing data, the density function, mean, and median survival time should be written in the aforementioned equations unconditionally.

9. Why we have survival analysis to deal censored data if we impute them?

Author’s response:

Regards. Imputation can be used for a number of purposes.

1- Survival models are utilized when the *dependent* variable is censored itself.

If censoring is documented in other variables, it is required to impute the censored ones (See Reference 1 below). For example, in cancer patients, the dependent variable is survival time. However, disease recurrence time is an independent variable with interval censoring. As a result, censorship must be considered, and censored data should be imputed while analyzing this variable.

2- When we aim to employ a method other than survival analysis. For example, in the Structural Equation Modeling (SEM) and the Bayesian network, both of which need the imputation of censored data. In such circumstances, the censored data must be managed in some way in order to have complete the data.
3- When we wish to perform exploratory analysis.

Assume we want to use an independent t-test to compare the average survival time of men and women. Assuming that part of the survival times is censored, comparisons on incomplete survival times may yield incorrect results. The same is true for Analysis of Variance (ANOVA) (See Reference 2 below).

10. Figures are too messy and cannot be readable. Improve the presentation of figures.

Author’s response:

Many thanks

All plots were redesigned, and high-quality diagrams were created. To avoid losing the quality of the graphs, the output of the R program was saved as a pdf.

11. I tried to run the code but it did not work. Please recheck the code.

Author’s response:

Thanks.

The code and Breast cancer data have been uploaded.

GitHub Link for R codes:

<https://github.com/pshkhoei/Thesis-Paper-1>

12. Include effective size as the diagnostic tool.

Author’s response:

Thanks a lot. Done.

13. Still I cannot figured out how the Bayesian analysis is done?

Author’s response:

Thankful.

The following steps are taken.

1- A distribution is assigned to the desired censored variable (Weibull or Birnbaum-Sanders).

2-The prior distribution is assigned to the parameters of the distribution.

3- Under the Bayesian approach, the value of the parameters is updated 10,000 times using the likelihood function and prior distribution (the posterior distribution of the parameters ($p\left( \theta| D \right)$) is estimated).

4- With Equation (7) in the paper:

$$f\left( t | T\geq c, D \right)= \int f\left( t | T\geq c, \theta\right)p\left( \theta| D \right)d\theta$$

each value of the posterior parameter is entered into Equation (7), and one data point is generated for each censored data point. If the posterior parameters are estimated 10,000 times, the censored value is simulated 10,000 times.

5- The censored data is replaced with the mean or median of the simulated times to have complete data.

6- The desired graph or analysis is performed based on the entire set of data.

**References**

1.	Svahn C, Sysoev O. Selective Imputation of Covariates in High Dimensional Censored Data. Journal of Computational and Graphical Statistics. 2022;31(4):1397-405.

2.	Fan J. Imputation Based Statistical Test for Right Censored Data. Kentucky: University of Louisville; 2007.

**Reviewer #2**

1. References are not correctly cited.

Author’s response:

Thanks.

All of the references were double-checked, and any necessary changes were made.

2.Equations are also not written properly. For example, the median survival equation.

Author’s response:

Many thanks.

The article has been thoroughly reviewed and modified. Grammar and spelling mistakes were fixed.

3. The authors must give comprehensive details on how the scale parameter is determined and how the values of X are generated.

Author’s response:

Thanks a lot.

There are two possible states for the variable $x$: zero and one, with equal probability of 0.5. This variable is used in the scale parameter of the distributions of the survival time (Weibull and Birnbaum-Saunders). That is, instead of using a fixed values (say 4) for the scale parameter, the scale was linked to a two-state variable which represented two groups.

4. Weibull must be written with capital W.

Author's response:

Thanks a lot. Done

5. Why do we have survival analysis to deal censored data if we impute them?

Author’s response:

Regards. Imputation can be used for a number of purposes.

1- Survival models are utilized when the *dependent* variable is censored itself.

If censoring is documented in other variables, it is required to impute the censored ones (See Reference 1 below). For example, in cancer patients, the dependent variable is survival time. However, disease recurrence time is an independent variable with interval censoring. As a result, censorship must be considered, and censored data should be imputed while analyzing this variable.

2- When we aim to employ a method other than survival analysis. For example, in the Structural Equation Modeling (SEM) and the Bayesian network, both of which need the imputation of censored data. In such circumstances, the censored data must be managed in some way in order to have complete the data.
3- When we wish to perform exploratory analysis.

Assume we want to use an independent t-test to compare the average survival time of men and women. Assuming that part of the survival times is censored, comparisons on incomplete survival times may yield incorrect results. The same is true for Analysis of Variance (ANOVA) (See Reference 2 below).

6. Figures are not readable.

Author’s response:

Many thanks.

All plots were redesigned, and high-quality diagrams were created. To avoid losing the quality of the graphs, the output of the R program was saved as a pdf.

7. Why gamma distribution is used as a prior?

Author's response:

Many thanks. Other distributions can also be used for prior distribution of parameters. In this study, the gamma distribution is used and its parameters are chosen such that we have a *reference prior* to provide a common base to evaluate data, rather than specific prior information.

8. How the DIC values are computed from the MCMC output?

Author's response:

Regards. An example is provided to clarify this question.

We define $y$ as having a normal distribution with a mean ($\theta)$ and a precision parameter ($\tau$).

$$y_{1}, \ldots, y_{n}|\theta\sim N\left( \theta, \frac{1}{\tau^{*}} \right),$$

For the desired parameter, the likelihood function and prior distribution are represented as $L(\theta|y)\propto exp(-\frac{n\tau^{*}}{2}\left( \bar{y}-\theta\right)^{2}),$ and $\theta\sim N(\theta_{0}, \frac{1}{\tau_{0}})$, respectively, and the posterior distribution of the parameter is also expressed as follows.

$$N(\frac{\tau_{0}}{\tau_{0}+n\tau_{*}} \theta_{0}+ \frac{n\tau_{*}}{\tau_{0}+n\tau_{*}}\bar{y.}, \frac{1}{\tau_{0}+n\tau_{*}})$$

Our task is to compute the DIC index.

$DIC=2 E\left( D\left( \theta| y \right) \right)-D(\hat{\theta)}$	*(1)*

As a result, $E\left( D\left( \theta| y \right) \right)$ and $D(\hat{\theta)}$ must be defined.

$D\left( \theta\right)=n\tau_{*}\left( \bar{y.}-\theta\right)^{2},$	*(2)*

$D\left( \hat{\theta} \right)=n\tau_{*}\left( \bar{y.}-\hat{\theta} \right)^{2}$	*(3)*

$\hat{\theta}=E(\theta|y)$	*(4)*

$D\left( \hat{\theta} \right)=n\tau_{*}\left( \bar{y.}-E(\theta|y \right)^{2}$	*(5)*

We must now compute $E(D\left( \theta| y \right)$.

$$E(D\left( \theta| y \right)=E\left( n\tau_{*}\left( \bar{y.}-\theta\right)^{2}|y \right)= n\tau_{*} E\left( \left( \bar{y.}-\theta\right)^{2} | y \right)= n\tau_{*}Var\left( \theta| y \right)+n\tau_{*} \left( E\left( \theta| y \right) \right)^{2}=$$

$n\tau_{*}Var\left( \theta| y \right)+n\tau_{*} \left( \hat{\theta}- \bar{y} \right)^{2}= \frac{n\tau_{*}}{n\tau_{*}+ \tau_{0}}+D\left( \hat{\theta} \right)= p_{D}+ D\left( \hat{\theta} \right)$	*(6)*

And we rewrite $D\left( \hat{\theta} \right)$ using equation 5.

$$D\left( \hat{\theta} \right)=n\tau_{*}\left( \bar{y.}-E(\theta|y \right)^{2}= n\tau_{*}\left( \bar{y.}-\left[ \frac{n\tau_{*}}{\tau_{0}+ n\tau_{*}}\bar{y.}+ \frac{\tau_{0}}{\tau_{0}+ n\tau_{*}}\theta_{0} \right] \right)^{2}=$$

$n\tau_{*}\left( \frac{\tau_{0}}{\tau_{0}+ n\tau_{*}} \right)^{2}{(\bar{y}. - \theta_{0})}^{2},$	*(7)*

The DIC index is expressed as follows using relations (1), (6), and (7).

$$DIC= 2 E\left( D\left( \theta| y \right) \right)-D(\hat{\theta)}=2 p_{D}+ 2 D\left( \hat{\theta} \right)- D\left( \hat{\theta} \right)= 2 p_{D}+ D\left( \hat{\theta} \right)=$$

$= 2 \frac{n\tau_{*}}{n\tau_{*}+ \tau_{0}}+ n\tau_{*}\left( \frac{\tau_{0}}{\tau_{0}+ n\tau_{*}} \right)^{2}{(\bar{y}. - \theta_{0})}^{2},$	*(8)*

Consequently, equation (8), and (3) is used to get the value of DIC (See Reference 3 below). The size of the DIC index is obtained by using the corresponding command code.

9. Some graphical assessment tools for MCMC convergence must be included.

Author's response:

Thanks a lot. I appreciate the point you raised. In this regard, the convergence of the simulations was evaluated using the Geweke’s diagnostics and is shown in graphs (6) and (7). The Geweke’s diagnostic Z-score indicate that the convergence is satisfied.

**References**

1.	Svahn C, Sysoev O. Selective Imputation of Covariates in High Dimensional Censored Data. Journal of Computational and Graphical Statistics. 2022;31(4):1397-405.

2.	Fan J. Imputation Based Statistical Test for Right Censored Data. Kentucky: University of Louisville; 2007.

3.	Christensen, R., Johnson, W., Branscum, A., & Hanson, T. E. (2010). Bayesian ideas and data analysis: an introduction for scientists and statisticians. CRC press.
